# Time to resolution of symptoms and recovery after mild traumatic brain injury: protocol for a systematic review and meta-analysis

**DOI:** 10.1136/bmjopen-2023-082700

**Published:** 2024-07-15

**Authors:** Adrian Ngai, Owen Hibberd, Hamda Hassan, Daniel P Whitehouse, Sophie Richter, Virginia Newcombe

**Affiliations:** 1 School of Clinical Medicine, Cambridge University, Cambridge, UK; 2 Emergency and Urgent Care Research in Cambridge (EURECA), PACE Section, Department of Medicine, University of Cambridge, Cambridge, UK

**Keywords:** Neurological injury, Follow-Up Studies, Adult neurology, REHABILITATION MEDICINE

## Abstract

**Abstract:**

**Introduction:**

Mild traumatic brain injury (mTBI) is a leading cause of morbidity and mortality, with approximately 1 out of 200 people each year sustaining an mTBI in Europe. There is a growing awareness that recovery may take months or years. However, the exact time frame of recovery remains ill-defined in the literature. This systematic review aims to record the range of outcome measures used for mTBI and understand the time to recovery for different outcomes.

**Methods and analysis:**

This protocol complies with the Preferred Reporting Items for Systematic Review and Meta-Analysis Protocols guideline. A prespecified literature search for articles in the English language will be conducted from database inception to the date of searches using MEDLINE and EMBASE. A trial search was conducted on 5 October 2023 with refinement of the search criteria following this. For each study, screening of the title, abstract and full text, as well as data extraction, will be done by two reviewers, with an adjudicating third reviewer if required. The risk of bias will be assessed using the Cochrane risk of bias tool for clinical trials and the Newcastle Ottawa score for cohort studies. The primary outcome is the time to resolution of symptoms in mTBI patients who have a full recovery, using any validated outcome measure. Results will be categorised by symptom groups, including but not limited to post-concussive symptoms, mental health, functional recovery and health-related quality of life. For mTBI patients who do not recover, this review will also explore the time to the plateau of symptoms and the sequelae of these symptoms. Where possible, meta-analysis will be undertaken, with a narrative review undertaken when this is not possible. Subgroup analyses of patients aged over 64 years, and patients with repetitive head injury, are planned.

**Ethical review and dissemination:**

Ethical review is not required, as no original data will be collected. Results will be disseminated through peer-reviewed publications and academic conferences.

**PROSPERO registration number:**

CRD42023462797.

**STRENGTHS AND LIMITATIONS OF THIS STUDY**

- We aim to provide a comprehensive and rigorous systematic review of recovery profiles following mild traumatic brain injury (mTBI), informing clinicians on the expected recovery and identifying specific targets for further research on therapeutic intervention.
- We will examine a wide range of outcome measures, in keeping with the heterogenous nature of deficits clinically observed following mTBI.
- The search algorithm is open with wide inclusion criteria, allowing identification of a wide range of papers to best inform the reviews conclusions.
- The heterogeneity of the included studies may limit the ability for quantitative summary in the form of meta-analysis, with a qualitative summary anticipated for a wide section of the literature.

## Background

 Traumatic brain injury (TBI) is a leading cause of morbidity and mortality worldwide, with a large associated economic burden on the global healthcare system.^1 2^ Over 85% of TBI may be classified as mild (mTBI) according to consciousness level and neurological functioning.^3 4^ mTBI leads to a significant public health burden. Every year in Europe, approximately 1 out of 200 people are affected by mTBI.^5^ Further, this is likely an underestimate, with mTBIs often undiagnosed and unrecorded.^6 7^

After excluding a need for admission or neurosurgical intervention, current practice commonly is to discharge patients who have mTBI with head injury advice and no routine follow-up unless specific concerns arise.^8^ A widely held dogma is that the majority of patients with mTBI go on to make a full recovery.^8 9^ However, ‘mild’ TBI should not be underestimated, with this classification presenting somewhat of a misnomer.^9^ A significant proportion of mTBI patients will continue to experience substantial, life-changing problems that can last months to years, representing a significant individual burden for patients and families and also a wider public health burden.^2 8^ These symptoms can include severe fatigue, poor memory, headaches and mental health issues (including anxiety, depression and post-traumatic stress).^2^ Further evidence, largely observed in the field of sports-related concussions, has highlighted potential long-term complications with repetitive minor head injuries (two or more prior concussions).^10^,^12^ However, the translation of this away from sport to the wider world of mTBI is less established.

There is increasing evidence of ongoing symptoms following mTBI.^8 13 14^ High-quality evidence has emerged from several large observational studies, including CENTER-TBI, TRACK-TBI and UPFRONT, generally demonstrating that 30%–50% of patients demonstrate functional deficits 3 to 12 months following mTBI.^15^,^21^ However, studies collecting serial outcome measurements represent a smaller pool of evidence.^22^ Further, there is significant heterogeneity in the literature concerning outcome measurement tools, the timepoint of outcome measurement and the definition of mTBI.

There is a need to synthesise the current literature to establish the landscape of outcome measures used in mTBI recovery and summarise the temporal recovery profile across the variety of symptomatic outcomes a patient may experience. A greater understanding of this can aid in clinical discussion with patients at the time of injury over expected recovery, inform when to follow-up with patients with ongoing symptoms and help identify patient groups and time periods for further therapeutic studies.

The aims of systematic review and meta-analysis are

1. to explore the scope of outcome measures used in mTBI research and
2. to build a picture of the temporal recovery profile of patients who have sustained a mTBI.

## Methods

The review will be conducted following the Preferred Reporting Items for Systematic Reviews and Meta-Analyses (PRISMA) guidelines. This study has been registered on the Prospero database (CRD42023462797).

### Eligibility criteria

The eligibility criteria for the Population, Intervention, Comparison, Outcomes and Study Design are detailed in table 1. Eligibility criteria for inclusion will include observational and interventional studies, enrolling adults (age equal to or greater than 16) with mTBI and assessing patient recovery. To reduce the risk of chronological and selection bias associated with later recruitment, only studies recruiting patients within 8 weeks of injury or studies that assessed participants prior to sustaining an injury will be included. A wide range of mTBI definitions will be included:

- Presentation with a history of a head injury, Glasgow Coma Score 13 to 15.^2 23^
- American Congress of Rehabilitation Medicine, revised by the WHO definition published in 1993^24^ and revised in 2023^25^: Glasgow Coma Score 13–15 at 30 min post-injury or later on presentation to healthcare, and one or more of the following symptoms: up to 30 min loss of consciousness, up to 24 hours post-traumatic amnesia, impaired mental state after and temporally congruent with injury (confusion or disorientation) and/or transient neurological deficit.^25 26^
- Clinical records data definition: a documented Abbreviated Injury Severity Scale score of 2 for the head region.^27^
- An administrative data definition for surveillance or research: cases of mTBI were recognised if the patients were assigned certain diagnostic codes chosen by the authors to be consistent with the diagnosis of mTBI. These include the International Classification of Diseases’ ninth and tenth editions.^28^

**Table 1:** Population, Exposure, Comparison, Outcomes and Study Design (PECOS) strategy for inclusion and exclusion

PECOS strategy	Inclusion criteria	Exclusion criteria
P – Population	Aged 16 years or overSustained an injury consistent with mild traumatic brain injury (mTBI) within 8 weeks of recruitment or had assessment prior to sustaining an mTBI.	No follow-up data availableSeverity of traumatic brain injury is not specifiedNo mild TBI-specific data can be extracted<30 cases of mTBI
E – Exposure	Mechanism of injury consistent with mTBI	
C – Comparator	No specific comparatorNot limited to studies with a control group	N/A
O – Outcome	Any study that follows up the patient beyond the time of recruitment at least once and reports on one or more outcome measures	Outcome(s) not clearly stated
S – Study design	Systematic reviews and meta-analysesRandomised controlled trialsCohort studies	Cross-sectional studiesCase-control studiesCase seriesCase reportsQualitative studiesReview articles

All reported outcomes and outcome measures, including functional recovery, mental health, physical health symptoms and health-related quality of life, will be included. The reference list of review articles in the search will be screened for further references that meet the inclusion criteria.

The exclusion criteria for study type will be case-control studies, case series, case reports, qualitative studies, review articles and cross-sectional studies. Other exclusion criteria include paediatric studies or mixed populations where no separated results are reported for adults or children respectively, no follow-up data and no specification of TBI severity or separate results for mTBI patients specifically.

Where multiple articles report on the same outcome measure at the same time point for the same patient cohort or overlapping patient cohorts, only one article will be included, prioritising larger sample sizes and more recent publication dates.

### Information sources

A planned literature search for articles in the English language will be conducted from inception to the search date using Cochrane, MEDLINE and EMBASE. A trial search was conducted on 5 October 2023 with refinement of the search criteria following this.

### Search strategy

The search strategy will use MeSH terms and text words to capture studies relating to mTBI, concussion and outcomes. The search strategy can be found in table 2.

**Table 2:** Search strategy for a systematic review and meta-analysis exploring the time to recovery for adult patients with mild traumatic brain injury

	MEDLINE	EMBASE
#1	exp post-concussion syndrome/ OR exp brain concussion/ OR exp brain injuries/ OR expcraniocerebral trauma/ OR exp brain injuries, traumatic/	exp concussion/ OR exp brain injuries/ OR exp head injury/ OR exp traumatic brain injury/
#2	(‘MTBI’ OR ‘mild TBI’ OR ‘TBI’ OR ‘traumatic brain inj*’ OR ‘mild traumatic brain injur*’).ab,ti.	(‘MTBI’ OR ‘mild TBI’ OR ‘TBI’ OR ‘traumatic brain inj*’ OR ‘mild traumatic brain injur*’).ab,ti.
#3	exp symptom assessment/ OR exp post-concussion syndrome OR exp mental disorders/ OR exp neurologic manifestations/ OR exp depression/ OR exp dizziness/ OR exp vertigo/ OR exp sleep wake disorders/ OR exp headache/ OR exp post-traumatic headache/ OR exp headache disorders, secondary/ OR exp fatigue/ OR exp mental fatigue/ OR exp memory/ OR exp memory disorders/ OR exp irritable mood/ OR exp anxiety/ OR exp anxiety disorders/ OR exp patient health questionnaire/ OR exp Glasgow outcome scale/ OR exp dissociative disorders/ OR exp stress disorders, post-traumatic/ OR exp return to work/ OR exp ‘Memory and Learning Tests’/ OR exp functional status/ OR exp ‘Recovery of Function’/ OR exp cognition/ OR exp mental health/ OR exp social status/ OR exp disease progression/ OR exp ‘Quality of Life’/ OR exp prognosis/ OR exp treatment outcome/ OR exp patient reported outcome measures/	exp symptom assessment/ OR exp postconcussion syndrome/ OR exp behaviour disorder/ OR exp neurologic disease/ OR exp depression/ OR exp mood disorder/ OR exp dizziness/ OR exp vertigo/ OR exp sleep disorder/ OR exp headache/ OR exp posttraumatic headache/ OR exp secondary headache/ OR exp fatigue/ OR exp mental fatigue/ OR exp memory/ OR exp cognition/ OR exp memory disorders/ OR exp irritability/ OR exp anxiety/ OR exp patient health questionnaire/ OR exp Glasgow outcome scale/ OR exp dissociative disorder/ OR exp mental disease/ OR exp posttraumatic stress disorder/ OR exp return to work/ OR exp cognitive function test/ OR exp functional status/ OR exp cognition/ OR exp mental health/ OR exp social status/ OR exp disease exacerbation OR exp ‘quality of life’/ OR exp prognosis/ OR exp treatment outcome/ OR exp patient-reported outcome/
#4	(‘symptom*’ or ‘prognos*’ or ‘quality of life’ or ‘functional’ or ‘mortality’ or ‘GOSE’ or ‘Rivermead’ or ‘outcome*’).ab,ti.	(‘symptom*’ or ‘prognos*’ or ‘quality of life’ or ‘functional’ or ‘mortality’ or ‘GOSE’ or ‘Rivermead’ or ‘outcome*’).ab,ti.
#5	exp time factors/ OR exp chronology as topic/ OR exp follow up studies/	exp time factor/ OR exp chronology/ OR exp follow up/
#6	(‘chronology’ OR ‘time course’ OR ‘recovery’ OR ‘resolution’ OR ‘rehabilitation’).ab,ti.	(‘chronology’ OR ‘time course’ OR ‘recovery’ OR ‘resolution’ OR ‘rehabilitation’).ab,ti.
#7	exp pediatrics/ OR exp child/ OR exp infant/ OR exp schools/	exp pediatrics/ OR exp child/ OR exp infant/ OR exp school/
#8	(‘p?ediatric*’ OR ‘child*’ OR ‘infant*’).ab,ti.	(‘p?ediatric*’ OR ‘child*’ OR ‘infant*’).ab,ti.

mTBImild traumatic brain injuryTBItraumatic brain injury

### Study records

Study selection will be performed using the online tool Rayyan (https://www.rayyan.ai) to allow for the removal of duplicate articles and the initial screening of titles and abstracts. Each title and abstract will be reviewed independently by two reviewers.^29^ Two votes will be required to exclude a paper with disagreements solved through discussion or consultations with a third reviewer (principal investigator). For abstracts meeting inclusion criteria, full texts will be retrieved, and each full text will again be independently reviewed against the inclusion and exclusion criteria by two reviewers and an adjudicating third reviewer if required. The reason for study exclusion will be recorded during the full-text screening.

The review will be undertaken in two stages to establish the landscape of outcome measures used and to facilitate appropriate categorisation of outcome measures. The first stage will identify the outcome measures and study cohort characteristics reported across the studies. The second stage will synthesise the results of the outcome measures. A standardised data abstraction form will be created for each data extraction stage, piloted on at least five articles by at least two reviewers each, and the forms will be adjusted as required. Data will be extracted by two independent reviewers for each selected paper. The following data items will be extracted in stage 1:

1. Study design
2. Study setting
3. Sample size
4. Definition of mTBI
5. Patient demographics (age, gender, population type (military, sport, community etc), education level, employment status)
6. Mechanism of injury, including intentional violence
7. Premorbid conditions (mental health (including post-traumatic stress disorder, depression and anxiety), sleep disorders, substance/alcohol use disorder, migraine, attention deficit disorder, dementia, other neurological conditions and other medical co-morbidities)
8. Other factors that may influence outcome: workers compensation, medicolegal action
9. Location of recruitment (emergency department, primary care, hospital admission, sporting field etc)
10. Glasgow Coma Score
11. Presence of abnormal CT head (percentage attributable to TBI)
12. Presence (number, severity) of subjects with a prior history of TBI
13. Time of recruitment relative to time of injury
14. Timing(s) of outcome measured
15. Outcome measures collected
16. Whether symptom or neurocognitive validity measures were collected and the result of such measures

The exact data items recorded for stage 2 will depend on the range of outcome measures found in stage 1. This will include:

1. Method of recording outcome measure (ie, total score, binary thresholds, clinical diagnosis)
2. Definition of complete recovery
3. Proportion of patients with complete symptom resolution at each measured time point and for each outcome measure
4. Loss to follow-up at each time point
5. Definition of complete recovery

The authors will be contacted directly when information from the published manuscripts is unavailable.

### Outcomes and Prioritisation

The primary outcome of this systematic review and meta-analysis is the time to resolution of symptoms following mTBI. For mTBI patients who do not recover, this review will also explore the time to the plateau of symptoms and sequelae of these symptoms.

Data will be synthesised following PRISMA guidelines.^30^

### Assessment of bias and heterogeneity

Studies will be assessed clinically and methodologically (study design, comparability, outcome ascertainment and risk of bias). The risk of bias will be evaluated using the Cochrane risk of bias tool 2.0 for clinical trials and the Newcastle Ottawa Score for cohort studies.^31 32^ This will include an assessment of the impact of missing data on effect estimates.^33 34^ Two reviewers will independently assess each included study for bias. Publication bias and small-study effects will be assessed using funnel plots for the most commonly recorded outcomes for included RCTs. As funnel plots are challenging to interpret for observational studies, they will not be used in this case. However, the potential for bias, including reporting, will be included in the discussion.^24^ The Egger method to assess asymmetry will be used for outcomes with more than 10 individual studies.

For studies reporting on the same outcome, the magnitude of variation or heterogeneity between studies will be measured by the index of heterogeneity (I^2^ and its CIs).^35^ I^2^ values of 25%, 50% and 75% are assumed to represent low, medium and high heterogeneity, respectively. The significance of the heterogeneity will be determined by χ^2^ for Q statistics.

### Data synthesis

Data extracted from included studies will be presented in evidence tables or as a narrative summary. We will analyse studies with one and multiple outcome time points separately and then pool this data if appropriate. For statistical analyses, a p value less than 0.05 will be considered significant.

If data are too heterogeneous to pool, then narrative synthesis of elements will be performed. This will focus on the time to resolution of symptoms in patients who have a full recovery and time to the plateau of symptoms and sequelae in patients who do not recover. Tables of data extracted from each study will be presented.

### Data analysis

A descriptive analysis will be performed. If studies have sufficiently homogenous populations, exposures and outcomes data will be pooled and meta-analysis performed to calculate summary measures of effect. To allow for expected differences between studies, a random-effects model will be used.

For single-outcome time point data, a series of exploratory multivariate regression models will be used to understand the effects of participant demographics and premorbid/post-morbid factors on outcome. If the relationship is not parametric, other non-parametric methods of curve fitting will be used.

For studies with more than one outcome timepoint, the recovery curve for each study will be presented graphically. If the data is parametric, the equation will be reported, and splines will be used for non-parametric data.

### Subgroup analyses

Where possible, prespecified subgroup analysis will be undertaken for key groups where differential extent and rate of recovery may be expected. These groups have been chosen due to either prior literature supporting this (eg, older age,^36^ female sex,^24^ repetitive TBI^11 12 37^) and/or them being discrete populations which have special characteristics (eg, military/blast injuries and sports concussion).^2^ The planned subanalyses will therefore be patient age (65 years or over compared with younger adults), sex, patients who have sustained more than one head injury, military and/or blast injuries and sports concussion, including subconcussive injuries. Where appropriate, sensitivity analyses will be performed using studies which have collected validity measures.

### Sensitivity analysis

If there is significant heterogeneity, sensitivity analyses will be performed, with outlying studies removed. Studies at a high risk of bias will also be excluded, and the main conclusions will be based on studies at a low risk of bias.

### Patient and public involvement

This review has been discussed with a patient and public involvement and engagement (PPIE) focus group run in conjunction with the NIHR Brain Injury MedTech Co-operative, which informed its content, including ensuring that different outcome measures were included. A PPIE panel will be involved with the interpretation and dissemination of results.
