# Supplementary material for: Time to resolution of symptoms and recovery after mild traumatic brain injury: protocol for a systematic review and meta-analysis
Source: BMJ Open. 2024 Jul 15;14(7):e082700. doi: 10.1136/bmjopen-2023-082700 (PMC11253732; doi:10.1136/bmjopen-2023-082700)
Supplement: Supplementary file 1 [file bmjopen-2023-082700.reviewer_comments.pdf]

PEER REVIEW HISTORY

BMJ Open publishes all reviews undertaken for accepted manuscripts. Reviewers are asked to complete a checklist review form (**http://bmjopen.bmj.com/site/about/resources/checklist.pdf**) and are provided with free text boxes to elaborate on their assessment. These free text comments are reproduced below.

ARTICLE DETAILS

**TITLE (PROVISIONAL)**	Time to Resolution of Symptoms and Recovery after Mild Traumatic Brain Injury: Protocol for a Systematic Review and Meta-Analysis
**AUTHORS**	Ngai, Adrian; Hibberd, Owen; Hassan, Hamda; Whitehouse, Daniel; Richter, Sophie; Newcombe, Virginia

VERSION 1 – REVIEW

**REVIEWER**	Kenney, Kimbra<br>Walter Reed National Military Medical Center, Department of Neurology
**REVIEW RETURNED**	26-Mar-2024

**GENERAL COMMENTS**	This is a timely and well-designed systemic review/meta-analysis of time-to-recovery and outcome measures of mild traumatic brain injury (mTBI) or concussion whose results/conclusions should provide data for design of future outcome studies, including timepoint and length of outcome measures as well as aid clinicians in the clinical evaluation and management of acute mTBI/concussion. The protocol proposes to include a meta-analysis/review of 2, to date under-studied TBI population- geriatric (>64 years of age) and individuals with repetitive mTBI (though not repetitive subconcussive head injury, as implicated in chronic traumatic encephalopathy and contact sports). The authors will adhere closely to the PRISM-P guidelines for their meta-analysis.<br>This protocol manuscript will be enhanced with the addition/clarification/consideration of the following:<br>1) Add justification for the 2 sub-groups to be separately analyzed and how/why they are unique to the overall population. Additionally, consider several other important and likely distinct sub-group analyses (e.g. military or blast, subconcussive, female) and justify why they will not be separately analyzed in this review.<br>2) For the I/E criteria: Justify the requirement for enrollment in a study within 7 days. In the US, ~85-90% of all TBIs are mTBIs, and of those, only ~20% present to EDs or acute healthcare settings within the first week. Thus, the restriction of including only those mTBI patients who enroll in a study within a week will not be representative of the full community-acquired mTBI patients. Many of these do not seek medical care because they become asymptomatic or recover quickly. By not including those who enroll later in studies will likely bias your results to longer recovery periods for mTBI. This would also exclude several large, observational mTBI studies that enroll much later after TBI/mTBI (e.g. the DoD "15-year natural history of TBI study" or LIMBIC-CENC). Please discuss how you will factor those who do not present for medical care at all or enroll in studies within a week into your time length to recovery analysis.

	3) How will the review account for over/under reporting by study participants? Will you add to your exclusion criteria, any study that does not include any symptom validity measures to their analysis? If not, how will the team control for symptom validity in their outcomes analysis?<br>4) Likewise with RCTs, will you add to your exclusion criteria, any study that does not include double blinding or, because of the nature of the intervention, is unable to adequately blind the study population? If not, how will the team control for potential study team or participant bias in these studies, particularly vis-à-vis symptom resolution/treatment?<br>5) Review the wording of the ACRM criteria for mTBI in the manuscript; should it be written as "up to 30 min of LOC or up to 24 hours of PTA", rather than as stated "30 min of LOC or 24 hours of PTA"?<br>6) There is an inconsistency between the I/E criteria in the body of the manuscript and Table 1. The body does not mention the inclusion of mTBI patients enrolled in RCTs, but Table 1 suggests that individuals enrolled in RCTs with >30 mTBI patients will be included.<br>7) Data collected: The protocol manuscript does not state whether any pre-morbid or co-morbid demographic factors or conditions, especially ones that have previously been associated with time to recovery and/or outcomes, will be collected (e.g. PTSD or other anxiety disorders, depression or other psychologic disorders, sleep disorders, employment status, Substance/alcohol use disorder dxes, workman's compensation or medico-legal actions related to injury, education or history of other pre-morbid conditions such as migraine, attention deficit disorder (ADD), dementia, among others). Please justify why these will not be collected and discuss how you will factor them into your time to recovery analyses.<br>8) The analysis section is rather brief and could benefit from a more in-depth description. In particular, there is no description of the qualitative analysis that will be done. Please describe how missing data will be handled, including how slightly different clinical study outcomes will be harmonized (e.g. normal CT versus MRI) or different symptom surveys used.

VERSION 1 – AUTHOR RESPONSE

Reviewer: 1
Dr. Kimbra Kenney, Walter Reed National Military Medical Center, Uniformed Services University
Comments to the Author:
This is a timely and well-designed systemic review/meta-analysis of time-to-recovery and outcome measures of mild traumatic brain injury (mTBI) or concussion whose results/conclusions should provide data for design of future outcome studies, including timepoint and length of outcome measures as well as aid clinicians in the clinical evaluation and management of acute mTBI/concussion. The protocol proposes to include a meta-analysis/review of 2, to date under-studied TBI population- geriatric (>64 years of age) and individuals with repetitive mTBI (though not repetitive subconcussive head injury, as implicated in chronic traumatic encephalopathy and contact sports). The authors will adhere closely to the PRISM-P guidelines for their meta-analysis. This protocol manuscript will be enhanced with the addition/clarification/consideration of the following:

1) Add justification for the 2 sub-groups to be separately analyzed and how/why they are unique to the overall population. Additionally, consider several other important and likely distinct sub-group analyses (e.g. military or blast, subconcussive, female) and justify why they will not be separately analyzed in this review.

Thank you for this important point. We agree that age and repetitive TBI are not the only important subgroups after TBI where understanding if there is differential recovery rates and characteristics is important. We have rewritten this section to include more motivation and the above key categories.

2) For the I/E criteria: Justify the requirement for enrollment in a study within 7 days. In the US, ~85-90% of all TBIs are mTBIs, and of those, only ~20% present to EDs or acute healthcare settings within the first week. Thus, the restriction of including only those mTBI patients who enroll in a study within a week will not be representative of the full community-acquired mTBI patients. Many of these do not seek medical care because they become asymptomatic or recover quickly. By not including those who enroll later in studies will likely bias your results to longer recovery periods for mTBI. This would also exclude several large, observational mTBI studies that enroll much later after TBI/mTBI (e.g. the DoD "15-year natural history of TBI study" or LIMBIC-CENC). Please discuss how you will factor those who do not present for medical care at all or enroll in studies within a week into your time length to recovery analysis.

The original motivation was to include those recruited in the hyperacute phase to try and obtain as “clean” a population as possible and so avoid selection bias which may occur with later recruitment. However as pointed out this would likely miss patients who sustain a TBI and remain in the community. To mitigate this we have therefore increased the window to a subacute window of within 8 weeks of injury as this time period will enable many patients with ongoing symptoms to have sought care but is within the timeframe of outcome assessed in many studies (3 months). We have also allowed inclusion of studies that have assessed people prior to their injury and after which many important large studies including military based have. We feel this is justified as these studies have a well characterised baseline for which to compare recovery.

3) How will the review account for over/under reporting by study participants? Will you add to your exclusion criteria, any study that does not include any symptom validity measures to their analysis? If not, how will the team control for symptom validity in their outcomes analysis?

Under/over reporting symptoms is a difficult issue in mTBI and may add significant biases to the results. Indeed symptom validity is an important part of assessment especially for symptoms and neuropsychological tests but in our experience is often not performed. We plan to collect as a variable whether or not symptom or neurocognitive validity measures were collected. A sensitivity analysis will be performed with papers who use these measures. This has been added to the methods section.

4) Likewise with RCTs, will you add to your exclusion criteria, any study that does not include double blinding or, because of the nature of the intervention, is unable to adequately blind the

study population? If not, how will the team control for potential study team or participant bias in these studies, particularly vis-à-vis symptom resolution/treatment?

We agree that blinding is an important component when assessing the quality of RCTs. Given blinding is not always possible we will not specifically exclude these studies. However we will assess this during the risk of bias assessment. Studies with high risk of bias will be excluded from analysis with main conclusions made only on studies with low risk of bias.

5) Review the wording of the ACRM criteria for mTBI in the manuscript; should it be written as "up to 30 min of LOC or up to 24 hours of PTA", rather than as stated "30 min of LOC or 24 hours of PTA"?

We have clarified the wording of the ACRM criteria and provided reference to the old version (which is likely to be used in most included papers) and the new version from 2023.

6) There is an inconsistency between the I/E criteria in the body of the manuscript and Table 1. The body does not mention the inclusion of mTBI patients enrolled in RCTs, but Table 1 suggests that individuals enrolled in RCTs with >30 mTBI patients will be included.

We have amended Table 1 to be more reflective of the planned systematic review including observational studies.

7) Data collected: The protocol manuscript does not state whether any pre-morbid or co-morbid demographic factors or conditions, especially ones that have previously been associated with time to recovery and/or outcomes, will be collected (e.g. PTSD or other anxiety disorders, depression or other psychologic disorders, sleep disorders, employment status, Substance/alcohol use disorder dxes, workman's compensation or medico-legal actions related to injury, education or history of other pre-morbid conditions such as migraine, attention deficit disorder (ADD), dementia, among others). Please justify why these will not be collected and discuss how you will factor them into your time to recovery analyses.

We did not originally include these as the aim of this review is not prognostication. However on reflection we can see that collecting these is important to be able to understand the context of the results. We have added this to the data collection plan.

8) The analysis section is rather brief and could benefit from a more in-depth description. In particular, there is no description of the qualitative analysis that will be done. Please describe how missing data will be handled, including how slightly different clinical study outcomes will be harmonized (e.g. normal CT versus MRI) or different symptom surveys used.

We have significantly expanded the methods to explain in more detail the analysis plans.

Missing data is often a significant issue in TBI studies, and two of the authors have been part of systematic reviews addressing this (references 1 and 2 below). The impact of missing data on effect estimates will be assessed in the risk of bias assessment and used to inform their overall GRADE ratings of risk of bias and their interpretation of the results which is the recommended approach in references 3 and 4 below. If the data is appropriate to perform quantitative analyses multiple imputation and sensitivity analyses will be performed.

We will harmonise data where there are accepted methods to do so. Given patients may be CT negative and MRI positive makes harmonisation of these variables challenging to do robustly. Different symptom surveys are also difficult to harmonise beyond whether a subject meets criteria for post-concussion syndrome or not for example. Given this we would aim to perform any quantitative analysis on the most commonly reported outcome measures separately with data from other measures being available in tables. For mental health measures we will use the recommended cut-off for each specific measure to determine if a patient has significant sequelae or not. These approaches of course have limitations which will be discussed in the Systematic Review itself.

References:

1. Richter S, et al. Handling of Missing Outcome Data in Traumatic Brain Injury Research: A Systematic Review. J Neurotrauma. 2019 Oct 1;36(19):2743-2752. doi: 10.1089/neu.2018.6216. PMID: 31062649; PMCID: PMC6744946.
2. Richter S, et al. Study Design Features Associated with Patient Attrition in Studies of Traumatic Brain Injury: A Systematic Review. J Neurotrauma. 2020 Sep 1;37(17):1845-1853. doi: 10.1089/neu.2020.7000. PMID: 32345119.
3. Kahale LA, et al. Potential impact of missing outcome data on treatment effects in systematic reviews: imputation study. BMJ. 2020;370:m2898. doi: 10.1136/bmj.m2898. PMID: 32847800; PMCID: PMC7448113.
4. Kahale LA, et al. A guidance was developed to identify participants with missing outcome data in randomized controlled trials. J Clin Epidemiol. 2019;115:55-63. doi: 10.1016/j.jclinepi.2019.07.003. PMID: 31299357.

Reviewer: 1
Competing interests of Reviewer: I have no competing interests.

VERSION 2 – REVIEW

**REVIEWER**	Kenney, Kimbra<br>Walter Reed National Military Medical Center, Department of Neurology
**REVIEW RETURNED**	08-Jun-2024

**GENERAL COMMENTS**	Thank you to the study team for considering my comments/suggestions and incorporating them into their study methods and resubmitting the revised manuscript. Besides, a few very minor edits/misspellings (e.g. p2 the sentence, "After excluding a need for admission or neurosurgical intervention, current practice commonly is to discharge patients who have mTBI with head injury advice and no routine follow-up unless specific

	concerns.", may read more smoothly if the word "arise" is added to the end of the sentence), I have no further comments/suggestions.

VERSION 2 – AUTHOR RESPONSE

Thank you.

This has been proofread for spelling and grammatical errors.
